# Prevalence of resistance associated substitutions and efficacy of baseline resistance-guided chronic hepatitis C treatment in Spain from the GEHEP-004 cohort

**DOI:** 10.1371/journal.pone.0221231

**Published:** 2019-08-30

**Authors:** Ana Belén Pérez, Natalia Chueca, Juan Macías, Juan Antonio Pineda, Javier Salmerón, Antonio Rivero-Juárez, Carmen Hidalgo-Tenorio, María Dolores Espinosa, Francisco Téllez, Miguel Ángel Von-Wichmann, Mohamed Omar, Jesús Santos, José Hernández-Quero, José Joaquin Antón, Antonio Collado, Ana Belén Lozano, Miguel García-Deltoro, Marta Casado, Juan Manuel Pascasio, Aida Selfa, José Miguel Rosales, Alberto De la Iglesia, Juan Ignacio Arenas, Silvia García-Bujalance, María José Ríos, Enrique Bernal, Onofre Martínez, Antonio García-Herola, Mónica Vélez, Pilar Rincón, Federico García

**Affiliations:** 1 Clinical Microbiology Unit, University Hospital Reina Sofía, Instituto Maimónides de Investigación Biomédica de Córdoba (IMIBIC), Córdoba, Spain; 2 Clinical Microbiology Unit, University Hospital San Cecilio, Instituto de Investigacion Ibs. Granada, Granada, Spain; 3 Infectious Diseases Unit, University Hospital Nuestra Señora de Valme, Sevilla, Spain; 4 Hepatology Unit, University Hospital San Cecilio Granada, Instituto de Investigación Ibs. CIBERehd, Granada, Spain; 5 Infectious Diseases Unit, University Hospital Reina Sofía, Instituto Maimónides de Investigación Biomédica de Córdoba (IMIBIC), Universidad de Córdoba, Córdoba, Spain; 6 Infectious Diseases Unit, University Hospital Virgen de las Nieves, Granada, Spain; 7 Hepatology Unit, University Hospital Virgen de las Nieves, Granada, Spain; 8 Infectious Diseases Unit, Hospital Puerto Real, Puerto Real, Cádiz, Spain; 9 Infectious Diseases Unit, University Hospital Donostia, San Sebastián, Spain; 10 Infectious Diseases Unit, Complejo Hospitalario de Jaén, Jáen, Spain; 11 Infectious Diseases Unit, University Hospital Virgen de la Victoria, Málaga, Spain; 12 Infectious Diseases Unit, University Hospital San Cecilio, Granada, Spain; 13 Penitenciary Physician, Albolote Penitenciary, Albolote, Granada, Spain; 14 Infectious Diseases Unit, Complejo Hospitalario Torrecárdenas, Almería, Spain; 15 Infectious Diseases Unit, Hospital de Poniente, El Ejido, Almería, Spain; 16 Infectious Diseases Unit, Hospital General de Valencia, Valencia, Spain; 17 Hepatology Unit, Complejo Hospitalario Torrecárdenas, Almería, Spain; 18 Hepatology Unit, University Hospital Virgen del Rocío, Seville, Spain; 19 Hepatology Unit, Hospital Comarcal Santa Ana, Motril Granada, Spain; 20 Hepatology Unit, Hospital Costa del Sol, Marbella, Málaga, Spain; 21 Clinical Microbiology Unit, University Hospital Infanta Elena, Huelva, Spain; 22 Hepatology Unit, University Hospital Donostia, San Sebastián, Spain; 23 Clinical Microbiology Unit, University Hospital La Paz, Madrid, Spain; 24 Infectious Diseases Unit, University Hospital Virgen Macarena, Seville, Spain; 25 Infectious Diseases Unit, Hospital General Reina Sofía, Murcia, Spain; 26 Infectious Diseases Unit, University Hospital Santa Lucía, Cartagena, Spain; 27 Hepatology Unit, Hospital Marina Baixa, Vilajoyosa, Alicante, Spain; 28 Infectious Diseases Unit, Hospital General de La Palma, La Palma, Spain; Nihon University School of Medicine, JAPAN

## Abstract

Treatment guidelines differ in their recommendation to determine baseline resistance associated substitutions (RAS) before starting a first-line treatment with direct-acting antivirals (DAAs). Here we analyze the efficacy of DAA treatment with baseline RAS information. We conducted a prospective study involving 23 centers collaborating in the GEHEP-004 DAA resistance cohort. Baseline NS5A and NS3 RASs were studied by Sanger sequencing. After issuing a comprehensive resistance report, the treating physician decided the therapy, duration and ribavirin use. Sustained virological response (SVR12) data are available in 275 patients. Baseline NS5A RAS prevalence was between 4.3% and 26.8% according to genotype, and NS3 RASs prevalence (GT1a) was 6.3%. Overall, SVR12 was 97.8%. Amongst HCV-GT1a patients, 75.0% had >800,000 IU/ml and most of those that started grazoprevir/elbasvir were treated for 12 weeks. In genotype 3, NS5A Y93H was detected in 9 patients. 42.8% of the HCV-GT3 patients that started sofosbuvir/velpatasvir included ribavirin, although only 14.7% carried Y93H. The efficacy of baseline resistance-guided treatment in our cohort has been high across the most prevalent HCV genotypes in Spain. The duration of the grazoprevir/elbasvir treatment adhered mostly to AASLD/IDSA recommendations. In cirrhotic patients infected with GT-3 there has been a high use of ribavirin.

## Introduction

Hepatitis C infection is a leading cause of liver diseases, liver cirrhosis, and hepatocellular carcinoma. As of 2015, the Polaris Observatory estimated that, globally, about 71 million of individuals have viraemic HCV infections and need antiviral treatment [1]. Direct Acting Antiviral (DAA) combinations that are currently recommended as first line treatment of HCV infected patients allow achieving high sustained virological response (SVR) rates (>90–95%) for all HCV genotypes [2]. HCV genotype, liver disease severity and previous treatment experience are still considered key factors to select first line treatment regimens by international treatment guidelines [3–4].

Because of its lifecycle and high replication kinetics, HCV exists within the same host as a population of very closed but different viral-variants, known as “quasispecies” [5]. Eight HCV genotypes, and more than 80 subtypes have been described [6–8]. Worldwide, HCV GT-1, GT-2, and GT-3 are the most prevalent genotypes [9]. In Spain HCV GT4 is relatively prevalent, while a very low prevalence of HCV GT2, HCV GT5 and HCV GT6 has been reported [10]. In contrast to the great amount of information on HCV genotype distribution, little information is known about the prevalence of resistance associated substitutions (RAS) in the different genotypes. Most data come from the retrospective analysis of pooled phase II and III clinical trials including patients with HCV GT-1 [11–12] and HCV GT-3 [13–14].

Failure to achieve HCV cure is an infrequent event that can happen with all HCV genotypes. Even in the era of the new pangenotypic DAAs, cirrhosis and previous interferon exposure still play an important role on initial treatment decision-making (3,4). DAA failure may also be associated with the presence of HCV RASs especially in NS5A [15–16]. Although the RASs found when a patient does not achieve SVR12 are generally developed during treatment, in some patients they may pre-exist as naturally occurring variants. Given the rationale that a special impact of baseline RASs on the efficacy of DAAs has been described for GT-1a and GT-3 [11,12,17,18], current AASLD guidelines [3] recommend baseline RAS testing for GT-1a infected patients before starting the Grazoprevir-Elbasvir combination, to decide if a 12 week regimen without ribavirin may be used, and also for GT-3 cirrhotic patients before starting the Sofosbuvir-Velpatasvir combination, to decide if ribavirin must be added. The universal use of HCV resistance testing is limited by the lack of a validated, widely available, and easy to access assay. EASL guidelines [4] are “resistance free”, mainly because In Europe there has been no standardized commercial test to determine RASs, and baseline testing is a barrier for treatment initiation.

The information on the impact of baseline RASs testing on the outcome of first line DAA HCV treatment in real life is scarce. As a substudy of the GEHEP-004 cohort [19], a real life cohort of patients failing their first interferon DAA regimen running in Spain, here we invited the cohort participants to assess the prevalence of baseline RASs according to viral genotype and subtype and to evaluate their impact on first line DAA efficacy in daily clinical practice.

## Patients and methods

We conducted a prospective study in 22 hospitals and 1 penitentiary centre attending HCV infected patients across Spain. All individuals who were to start treatment with all-oral DAA-based regimens, who had never been treated before with DAAs, and with an available baseline RAS test in NS5A, and a comprehensive interpretation report were included. The treating clinician decided the DAA regimen, according to the DAAs available during the different phases of the study period (January 2015 to November 2017), as well as other factors as the severity of liver disease, interactions with concomitant medications, and comorbidities, following treatment guidelines [3,4,20]. Patients with RASs in NS5A were treated with a DAA combination containing a protease inhibitor when possible, or, alternatively, adding ribavirin and/or extending treatment.

Baseline genotyping was performed as a part of routine clinical care through the commercially available test at the participating centre (Versant HCV Genotype 2.0 LiPA assay, for the majority of the samples, but also the Abbott Real Time HCV Genotype II assay and Trugene HCV Genotyping Kit were used). Plasma viral load was evaluated at baseline and, at least, at week 12 post-treatment. SVR12 was defined as undetectable plasma HCV RNA 12 weeks after the end of therapy.

For resistance analysis we used Sanger sequencing of the NS5A region for GT 1a, 1b, 3 and 4, and NS3 region for GT1a. Briefly, after RNA extraction using the Magnapure compact system (Roche), we performed a random primed cDNA synthesis (ThermoScientific). cDNA was used for a primer specific or a pangenotypic amplification depending on the HCV gene and the geno/subtype, and sequenced on an ABI Prism 3500 analyzer. A detailed description of the primers, amplification, and sequencing reactions can be found elsewhere [21]. The HCV genotype and subtype of the samples was also determined from the NS5B sequence by manual phylogenetic analysis and the use of subtyping tools COMET and Oxford. When present, RAS were transformed into a comprehensive report for clinicians according to the interpretation guidelines of the consensus statement from Lontok et al [22]. NS5A RASs 28A/G/T, 30D/E/H/G/K/L/R, 31F/M/V and 93C/H/N/S were considered as Elbasvir specific RASs (4), and NS5A RASs 30K, 31M/P/V, 92 K, and 93H/N/R were considered as Velpatasvir specific RASs [23].

The study was designed and performed according to the Helsinki declaration and was approved by the local Ethics Committee of Hospital Universitario San Cecilio. Verbal informed consent was obtained from participants in the study.

The data underlying this study have been deposited to Figshare and are accessible via https://doi.org/10.6084/m9.figshare.8796359.v2. All other relevant data are within the paper.

## Results

A total of 516 HCV-infected patients were included in the study. Patients were mainly male (78.0%), with a median of 52 years of age (IQR 48–56), a median baseline HCV viral load of 6.31 Log IU/ml (IQR 5.77–6.74), 31.1% with cirrhosis and 24.6% had prior experience with interferon. Most of the patients were infected with HCV GT-1a (n = 223; 43.2%), followed by 141 patients with HCV GT-3 infection (27.3%), 82 patients with HCV GT-1b infection (15.9%), and 70 patients with HCV GT-4 infection (13.6%). The main characteristics of the study population are shown in Table 1.

**Table 1:** Baseline characteristics of the patients.

	n = 516
**Age, years (IQR)**	52 (IQR 48–56)
**Male gender, n (%)**	78.0%
**Cirrhotics, n (%)**	31.1%
**Pre-treated, n (%)**	24.6%
**HCV RNA (Log IU/ml) (IQR)**	6.31 (IQR 5.77–6.74)
**HCV genotype**	
**1a, n (%)**	223 (43.2%)
**1b, n (%)**	82 (15.9%)
**3, n (%)**	141 (27.3%)
**4, n (%)**	70 (13.6%)

Overall, 70 patients (13.6%) showed RASs in NS5A and/or NS3 (n = 56, 10.8%, for NS5A; n = 14, 6.3%; for NS3). For GT1a, 36 patients (16.2%) showed RASs in NS5A and/or NS3 (n = 22, 9.9%, for NS5A; n = 14, 6.3%; for NS3). Twenty-two (26.8%) patients infected with GT1b, 9 (6.4%) patients infected with GT 3, and 3 (4.3%) patients infected with GT 4 harboured RASs in NS5A. A detailed description of the RASs in NS5A for HCV GT1a, 1b, 3 and 4, and for also RASs in NS3 for GT1a is shown in Table 2.

**Table 2:** Prevalence of the resistance associated substitutions (RASs) in NS5a/NS3 in the population studied.

	Position	GT 1a (n; %)	GT 1b (n; %)	GT 3 (n; %)	GT 4 (n; %)
NS5A	24	-	-	-	-
	28	11; 4.9%	-	-	2; 2.8%
	30	8; 3.6%	1; 1.2%	9; 6.4% 9; 6.4%* (A30MSV)	1; 1.4%
	31	3; 1.3%	12; 14.6%	-	3; 4.3%* (M31LV)
	32	1; 0.4%* (P32H)	-	-	-
	38	-	-	-	-
	58	1; 0.4% 14; 6.3%* (H58GPY)	3; 3.6% 3; 3.6%* (P58AT)	-	1; 1.4%* (P58L)
	62	-	-	-	-
	92	-	2; 2.4%* (A92T)	-	-
	93	1; 0.4%* (Y93L)	6; 7.3%	9; 6.4%	-
NS3	36	1; 0.4% 3; 1.3%* (V36L)			
	54	-			
	55	1; 0.4%* (V55A)			
	56	-			
	80	6; 2.7% 2; 0.9%* (Q80L)			
	122	5; 2.2% 3; 1.3%* (S122N)			
	168	2; 0.9%			
	170	-			

* in bold: these changes are not listed in Lontok´s consensus statement

**Fig 1:**
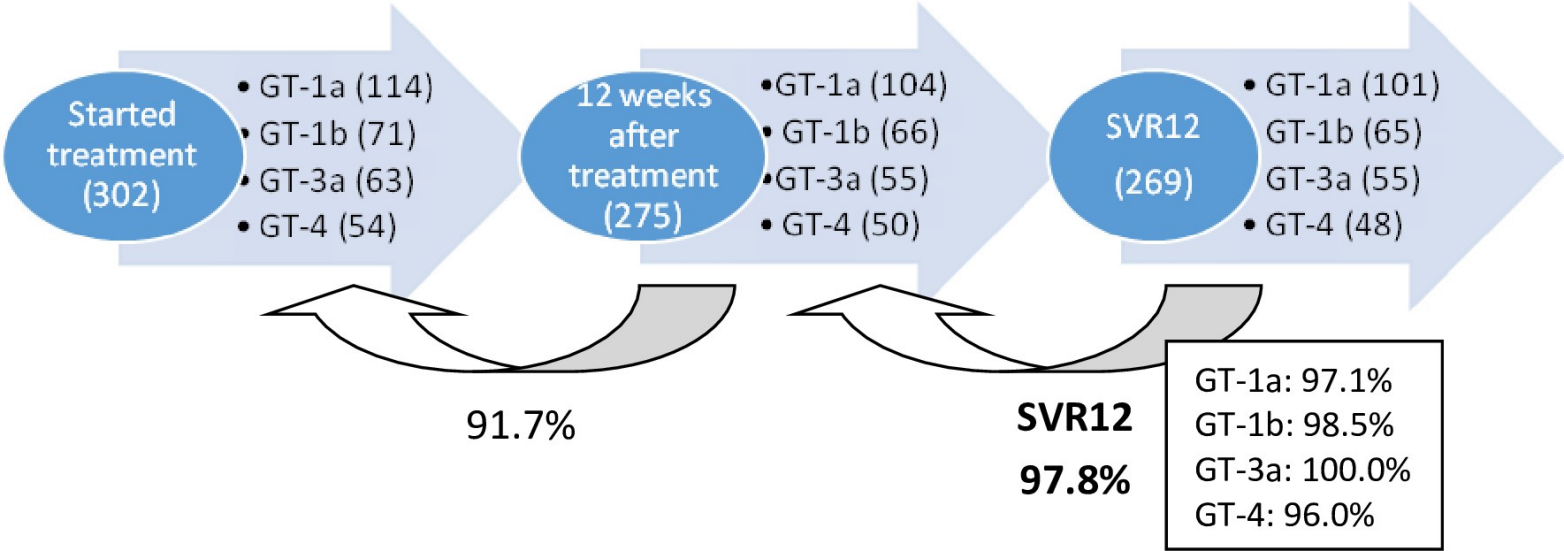
Overall and genotype-dependent efficacy of DAA treatment (mITT analysis).

Three hundred and two patients started DAA treatment. A detailed description of the regimens that the patients have started can be seen in Table 3. By the time of analysis, 285/302 patients (94.4%) have reached end of treatment (EOT) and SVR12 data is available for 275 patients. Four patients were lost to follow up (LTFU) due to premature interruption because of adverse events, 11 interrupted treatment voluntarily, 1 patient was LTFU by change of location, 8 reached EOT but were LTFU before evaluating SVR12 and, 1 patient died seven days before EOT. On a modified intention to treat analysis (mITT), 97.8% achieved SVR12. By genotype, all the GT3 infected patients and 98.5% of the GT1b infected patients achieved SVR12, while GT1a and GT4 achieved 97.1% and 96.0% SVR12 respectively. These data are represented in Fig 1. Six relapses were observed (3 patients infected by GT1a, 1 GT1b and 2 GT4). None of them were cirrhotic and three were treatment experienced. All of them had initiated a DAA regimen recommended by treatment guidelines. All patients with baseline RASs achieved SVR12.

**Table 3:** DAA regimens used according to HCV genotype, cirrhosis and previous treatment experience (TE).

HCV Genotype	DAA régimen (n)	Cirrhosis (n/N; %)	TE (n/N; %)	Cirrhosis & TE (n/N; %)
**1a**	SOF-LDV±RBV (79)	16/78; 20.5%	21/74; 28.4%	7/73; 9.6%
	SOF-SMV-RBV (1)	-	1/1; 100.0%	-
	PrOD±RBV (20)	1/19; 5.3%	4/15; 26.7%	-
	GRZ-EBV±RBV (13)	2/13; 15.4%	2/11; 18.2%	-
	SOF-VEL (1)	1/1; 100.0%	-	-
**1b**	SOF-LDV±RBV (35)	6/35; 17.1%	4/35; 11.4%	3/35; 8.6%
	SOF-SMV±RBV (2)	-	-	-
	PrOD±RBV (33)	10/32; 31.2%	10/33; 30.3%	1/32; 3.1%
	GRZ-EBV (1)	1/1; 100.0%	-	-
**3**	SOF-PegINT-RBV (2)	-	-	-
	SOF-DCV±RBV (45)	8/44; 18.2%	6/44; 13.6%	4/43; 9.3%
	SOF-LDV-RBV (2)	1/2; 50.0%	-	1/2; 50.0%
	SOF-VEL±RBV (14)	8/14; 57.1%	-	3/14; 21.4%
**4**	SOF-LDV±RBV (25)	7/25; 28.0%	4/25; 16.0%	1/25; 4.0%
	SOF-DCV (1)	-	-	-
	SOF-SMV (2)	-	-	-
	PrO±RBV (22)	5/21; 23.8%	2/22; 9.1%	1/21; 4.7%
	GRZ-EBV (3)	-	1/3; 33.3%	-
	SOF-GRZ-EBV (1)	-	-	-

SOF = Sofosbuvir; LDV = Ledipasvir; RBV = Ribavirine; SMV = Simeprevir, PrOD = Paritaprevir/_ritonavir_/Ombitasvir/Dasabuvir; GRZ = Grazoprevir

PrO = Paritaprevir/_ritonavir_/Ombitasvir; EBV = Elbasvir; VEL = Velpatasvir; PegINT = Pegylated Interferon; DCV = Daclatasvir.

For the genotype 1a infected patients we conducted a specific analysis on Elbasvir RASs. In contrast to NS5A class RASs that were detected in 22 (9.8%) of the GT1a infected patients, Elbasvir specific RASs were detected in only 12 patients; 5.4% [M28T (n = 1), Q30DEHR (n = 8), L31M (n = 3)]. Similar differences from NS5a class RASs to Elbasvir specific RASs were observed in the cirrhotic population: 9.5% had specific Elbasvir RASs, while 14.3% had NS5A class RASs. Interestingly, 158/210 patients had a viral load higher than 800.000 UI/ml and only 8 (5.1%) harboured Elbasvir RASs. Thirteen patients infected by GT1a in the study have started a Grazoprevir-Elbasvir baseline RAS guided treatment, two of them added ribavirin to the regimen and, only three patients were put on a 16-week regimen irrespective of the baseline viral load. All of these patients have achieved SVR12 except one that was LTFU. The characteristics of GT1a infected patients who have started Grazoprevir-Elbasvir baseline RAS guided treatment are shown in Table 4.

**Table 4:** Characteristics of GT1a infected patients who have started Grazoprevir-Elbasvir baseline RAS guided treatment.

Patient	Viral load (Log IU/ml)	Cirrhosis	Baseline RAS	Duration (weeks)	RBV	SVR12
1	6.29 (>800,000 IU/ml)	No	none	16 w.	Yes	Yes
2	6.52 (>800,000 IU/ml)	Yes	none	16 w.	Yes	Yes
3	6.28 (>800,000 IU/ml)	No	none	12 w.	No	Yes
4	6.06 (>800,000 IU/ml)	No	none	12 w.	No	No (LTFU)
5	7.02 (>800,000 IU/ml)	No	none	16 w.	No	Yes
6	6.59 (>800,000 IU/ml)	Yes	none	12 w.	No	Yes
7	6.52 (>800,000 IU/ml)	No	none	12 w.	No	Yes
8	6.29 (>800,000 IU/ml)	No	none	12 w.	No	Yes
9	6.90 (>800,000 IU/ml)	No	none	12 w.	No	Yes
10	6.47 (>800,000 IU/ml)	No	none	12 w.	No	Yes
11	5.65 (<800,000 IU/ml)	No	none	12 w.	No	Yes
12	6.60 (>800,000 IU/ml)	No	none	12 w.	No	Yes
13	6.66 (>800,000 IU/ml)	No	none	12 w.	No	Yes

LTFU: lost to follow-up.

In a similar way, we conducted a specific analysis on Velpatasvir RASs for the genotype 3 infected patients. In contrast to NS5A class RASs that were detected in 6.4% of the patients (n = 9), Velpatasvir specific RASs were detected in 18 patients; 12.7% [A30K (n = 9), Y93H (n = 9)]. Similar differences from NS5a class RASs to Velpatasvir specific RASs were observed in the cirrhotic population: 9/58 cirrhotic patients (15% of the cirrhotic population) had Y93H, and 14/58 cirrhotic patients (24% of the cirrhotic population) had specific Velpatasvir RASs. Fourteen cirrhotic patients infected by GT3 in the study have started a Sofosbuvir-Velpatasvir baseline RAS guided treatment, and 6 of them (42.8%) added Ribavirine to the regimen, but only 2 showed Y93H at baseline. All of these 14 patients have achieved SVR12 except one patient who was LTFU.

## Discussion

Baseline resistance testing prior to HCV treatment initiation with all oral DAA regimens is still a matter of debate, at least for certain scenarios. Although there is a full consensus that there is no need for baseline RAS testing in patients infected by genotypes 1b and 4, clinical treatment guidelines [3,4] still differ in recommendations for patients infected by genotypes 1a and 3. In our study we have observed very high efficacy rates of a baseline resistance-guided treatment initiation across all the most prevalent HCV genotypes in Spain. Interestingly the lowest SVR12 rates were found for genotype 4 (96%) and the highest for genotype 3 (100%) infected patients.

Patients infected by genotypes 1b (16%) and 4 (14%) were the least prevalent in our cohort. In fact, this is one of the limitations of our study, as this prevalence is not representative of GT 1b and 4 in Spain [10] and Europe [24]. These patients were also from the initial phases of our study, when the clinical role of baseline resistance testing was not restricted to genotypes 1a and 3. Nevertheless, only one patient infected by genotype 1b, and two patients infected by genotype 4, did not achieve SVR12 in our cohort [SVR12 98.5% (65/66) and 96% (48/50), respectively].

Our study also depicts the prevalence of NS5A RASs across HCV genotypes in Spain, and confirms, as previous studies [11] that genotype 1b harbours the highest rates of RAS prevalence (28%). Genotype 4 was the HCV-GT with the lowest rate of detection of clinically relevant RASs (3%). Of note, this data may be biased by the fact that we used the 2015 Lontok consensus statement for the evaluation of clinically significant RASs and by this time the knowledge on GT4 RASs was scarce.

Genotype 1a is the second genotype in prevalence in Spain [10], but it was the most prevalent in our cohort. This allowed us to generate very robust data on both, the prevalence of clinically relevant RASs across Spanish HCV GT 1a isolates, and baseline resistance-guided efficacy of HCV GT1a first line treatment. As Grazoprevir/Elbasvir was approved for GT1a treatment during the study period, we were also able to generate interesting data on its use in Spain, and how clinicians adhered to European or AASLD guidance. As for a prior study that describes RASs prevalence in patients from clinical trials in Europe [11], the prevalence of RASs in genotype 1a was low in our cohort: only 22 patients (9.9%) had any of the NS5A and 14 (6.3%) any of the NS3 RASs of clinical interest described in the Lontok consensus. As for clinical trial data [12], interestingly, in our real-life cohort the number of patients harbouring any Elbasvir specific RAS was even lower (n = 12, 5.4%). These finding had a high impact on the use of Grazoprevir/Elbasvir that, in our cohort, followed mostly the AASLD guidelines rather than the EASL ones. In fact, while 75% of the genotype 1a patients in our cohort had a viral load higher than 800.000 IU/ml, only 27% of the patients starting Grazoprevir/Elbasvir went on a 16-week plus Ribavirine regimen. Baseline resistance-guided efficacy was very high in our real-life genotype 1a cohort: SVR12 was achieved in 101/104 patients (97.1%). Real-life data for baseline resistance-guided efficacy are scarce. Cento et al [25] described a 100% SVR rate-using baseline RASs guided treatment for a small subset of genotype 1a infected patients in clinical practice.

Patients infected by genotype 3 were also overrepresented in our cohort (27%). We believe that the influence of AASLD clinical guidelines, which recommend baseline RAS testing for cirrhotic patients infected by genotype 3, in order to decide if adding Ribavirine to the Sofosbuvir/Vepatasvir combination is necessary, was responsible for the inclusion of this high number of genotype 3 infected patients in our cohort. Again, this also allowed us to generate robust data on the prevalence of clinically relevant RASs across Spanish HCV GT 3 isolates, and baseline resistance-guided efficacy of HCV GT3 first line treatment. As for the genotype 1a NS5A class RASs and Elbasvir, in genotype 3 again the prevalence of NS5A class RASs was higher (n = 27, 19%) than that of specific Velpatasvir RASs. Interestingly, only 9 patients (6%) carried the Y93H NS5A RAS at baseline, with an increase to 15% for the cirrhotic GT3 cohort. These numbers are slightly higher than that described by others in Spain [26] and Europe [27]. We also found that another 9 patients carried changes A30KS in NS5A. These changes, especially A30K, have a high impact on the efficacy of Glecaprevir/Pibrentasvir [28–29]. In fact, the most recent Spanish Guidelines [20,30] recommend a 12-week duration of G/P if these variants are present at baseline in genotype 3. As in the Cento et al [25] study, here we describe a 100% efficacy for our real-life baseline resistance-guided genotype 3 cohort: SVR12 was achieved in all of the 55 patients that were available for SVR12 evaluation, even if 39.6% (21/53) of these genotype 3 infected patients were cirrhotic.

In addition to the limitations we have already described, our study has some others: firstly, the design of the study, being a single-arm with no comparative arm without resistance information. Secondly, not all the patients that had a baseline resistance test started treatment; however, as restrictions to prioritize treatment for F3-F4 patients were available during the most time of the study period, we believe that the less favourable patients have been included here. Finally, and most importantly, even if we present here one of the most numerous real-life cohorts addressing the impact of baseline resistance-guided treatment, we believe that the number of patients included for each genotype may be too small to draw definitive conclusions, and we claim for international real-life cohorts collaborations to finally end this debate.
